# Transcranial Route of Brain Targeted Delivery of Methadone in Oil

**DOI:** 10.4103/0250-474X.56024

**Published:** 2009

**Authors:** W. Pathirana, P. Abhayawardhana, H. Kariyawasam, W. D. Ratnasooriya

**Affiliations:** Department of Pharmacology and Pharmacy, Faculty of Medicine, University of Colombo, Kynsey Road, Colombo 8, Sri Lanka; 1 Department of Zoology, Faculty of Science, University of Colombo, Colombo 3, Sri Lanka

**Keywords:** Transcranial route (TCR), emissary veins, intracranial sinuses, catchment area, methadone base, brain targeted

## Abstract

The unique anatomical arrangement of blood vessels and sinuses in the human skull and the brain, the prevalence of a high density of skin appendages in the scalp, extracranial vessels of the scalp communicating with the brain via emissary veins and most importantly, the way that the scalp is used in *Ayurvedic* medical system in treating diseases associated with the brain show that a drug could be transcranially delivered and targeted to the brain through the scalp. The present study was to investigate by measuring the antinociceptive effect on rats whether the opioid analgesic methadone could be delivered and targeted to the brain by transcranial delivery route. A non aqueous solution of methadone base in sesame oil was used for the application on the scalp. Animal studies were carried out using six groups of male rats consisting of group 1, the oral control treated with distilled water 1 ml; group 2, the oral positive control treated with methadone hydrochloride solution 316.5 μg/ml; group 3, the negative control treated transcranially with the blank sesame oil 0.2 ml and three test groups 4, 5 and 6 treated with three different dose levels of the transcranial oil formulation of methadone base, 41.6 μg/0.2 ml, 104 μg/0.2 ml and 208 μg/0.2 ml, respectively. The antinociceptive effects were examined by subjecting the rats to the hot plate and tail flick tests. The two higher concentrations of the three transcranial methadone formulations yielded response vs time curves showing nearly equal maximum antinociceptive effects similar to that of the oral positive control. Maximum analgesic effect after transcranial administration was observed between 1st and 2nd h and declined up to 6th hour. The results indicate that the transcranial brain targeted delivery of methadone base in the form of an oil based non aqueous solution results in statistically significant antinociceptive effects under experimental conditions. Therefore, it is possible to deliver central nervous system drugs through the proposed transcranial route when suitably formulated.

Apart from the established routes of drug administration practiced in modern medicine, *Ayurvedic* system of medicine uses a special route which involves oil therapies to the head. These therapies are used for centuries to treat diseases of the central nervous system[1]. The drug delivery route under taken in the present study is incidental to a special anatomical feature of the skull. The emissary veins draining blood from extracranial sites into the intracranial sinuses pierce a series of foramina present in the cranial bones. Scalp veins communicate with the sinuses of the brain via emissary veins. There are thirteen emissary veins connecting extracranial sites of the head with intracranial sinuses[2]. Seven major sinuses within the skull are inter connected by a number of anastomosing veins, which finally drain intracranially into jugular veins giving ample scope for the diffusion of the drug molecules into the nerve tissue of the brain. These anatomical arrangements of the vascular system of the brain are made use of in the investigations to establish the brain targeted transcranial route (TCR) of drug delivery.

TCR describes the passage of an oil solubilized drug moiety across the skin of the scalp including appendages of the skin such as sebaceous glands, walls of the hair follicles and sweat glands, through the cranial bones along with the diploe, the cranial bone sutures, the meninges and specifically through the emissary veins into the brain. It has to be noted that the total surface area of the skin along with the skin appendages adds up to a much larger area against the immediately visible plain skin surface in the scalp where the hair follicle density is very high compared to the rest of the skin. The initial vigorous ‘rubbing in’ of the medicated oil is an essential part of the transcranial drug administration in order to bring the oil into intimate contact with the epithelium of the skin appendages. The effective catchment area for the proposed transcranial route is considered to be the area of the head lying above the contour drawn through the four points consisting of two corners of the mouth and the two ears in the case of all higher animals[3]. An earlier study on transcranial route carried out with diazepam in sesame oil in which the centrally mediated muscle relaxant effect was studied by rotarod measurements has yielded positive results[4]. In the present study, central effects of methadone on rats were evaluated by hot plate and tail flick screening tests over a period of 6 h. The rotarod, hot plate and tail flick experimental measurements involving diazepam and methadone taken together could establish the possibility of transcranial drug delivery by the proposed transcranial route.

The oil therapies of *Ayurveda* using the head include *Shirodara, Shiroabyanga, Shiropitchu, Shirovasthi* and *Shiropralepa* in which drugs are delivered by the transcranial route[5]. Most of these *Ayurvedic* preparations are oil based. An important dosage design feature in the present study is the use of an essentially non-polar active drug moiety methadone base dissolved in an oil medium as against the use of the polar salt forms dissolved in aqueous media that are popular with modern formulations.

## MATERIALS AND METHODS

### Oral methadone dose for rats:

Methadone hydrochloride 5 mg tablets were reformulated to a rat dose to be administered in 1 ml of an aqueous medium for testing the oral positive control group 2. It was calculated considering the adult human methadone hydrochloride dose as 10 mg for a 60 kg adult and adopting it to 189.87 g, which was the average weight of the rats in groups 1 and 2. This value was increased ten fold considering the high metabolic rate in rats as against man. The amount of methadone for the preparation of 10 ml of the solution is therefore expressed as (10×189.87×10×10)/(60×1000) = 3.165 mg. An accurately weighed quantity of the powdered tablet equivalent to 3.165 mg of methadone was dissolved in 10 ml of distilled water immediately before administration of the oral dose of 316.5 μg/ml of methadone.

### Extraction of methadone base and preparation of transcranial sesame oil formulation:

The methadone base was extracted from methadone hydrochloride 5 mg tablets since this active ingredient is difficult to procure. Twenty tablets were powdered, dissolved in 10 ml of distilled water and the solution was centrifuged at 3000 rpm for 15 min. The supernatant was collected into a separating funnel and it was made alkaline to litmus with 20% w/v NaOH solution. The precipitate was extracted with two 5 ml portions of diethyl ether and it was washed with 10 ml of distilled water. The ether layer was dried over anhydrous MgSO_4_ and was evaporated to obtain the methadone base.

Based on the average human methadone hydrochloride dose of 10 mg for a 60 kg adult, the rat dose was calculated for a 200 g average weight rat, which is the near approximation of the weights of the rats in the groups 3, 4, 5 and 6. The above quantity was increased five fold for the transcranial application as against the oral dose since preliminary trials indicated that a simple adoption of the oral dose is insufficient for the transcranial route. It was once again increased ten times to compensate for the high rate of metabolism in rats. However, since the brain targeted dose is expected to concentrate in the brain the above dose was divided by 8 which was considered to be the fraction of the weight of the brain against the total weight of the rat. The final transcranial dose had to be delivered in a 0.2 ml volume as it is the amount that could be applied on the restricted scalp area of the rat without undue spillage. Therefore, based on the adult human dose, the transcranial dose in 0.2 ml of the oil for a 200 g rat can be expressed as (10×200×5×10×1000)/(60×1000×8) = 208 μg. The amount for 5 ml of the solution is therefore (208×5)/(1000×0.2) = 5.2 mg methadone base. This amount was dissolved in 5 ml of sesame oil on the day before the experiments. The product was left overnight until a clear solution was formed to give a 208 μg/0.2 ml stock solution after complete dissolution. A portion of the stock solution was diluted with sesame oil to twice its volume and a second portion was diluted to five times its volume to produce the mid test dose of 104 μg/0.2 ml and the low test dose of 41.6 μg/0.2 ml solutions, respectively.

### Laboratory animals for transcranial drug delivery experiments:

Ethical clearance for animal experiments was granted by the Ethics Review Committee of the Medical Faculty, University of Colombo. Healthy adult inbred male Sprague Dawley rats weighing 186 g to 205 g were used in the study. Animals were housed in a room maintained between 28°-31° with an alternating 12 h light/dark cycle with free access to pelleted food and water. Apart from experimental procedures, the animals were handled only during cage cleaning. They were acclimatized to laboratory conditions before experiments were carried out. Animals were assigned into six groups consisting of six animals each.

### Method of transcranial drug administration:

The hair of the scalp of animals in groups 3, 4, 5 and 6 was trimmed without injuring the skin. This was done within the trapezoidal area of the head bound by the pair of eyes and ears. Each rat in these four groups was treated with 0.2 ml of the respective oil application in 1/3 quantities by applying drop wise on to the hair trimmed bald area of the scalp in three stages as follows. First 1/3^rd^ application at 00:00 time, second 1/3^rd^ application at 00:15 minutes and third 1/3^rd^ application at 00:30 min. Each application was followed by ‘rubbing in’ for 2 min with an electric scalp stroking device consisting of an applicator 8×8 mm, attached to a 7 cm arm having a frequency of 4 Hz. This was to facilitate dispelling any air pockets and to bring the oil solution into intimate contact with the skin and its appendages of the scalp. Sixty minutes after the application of the first 1/3^rd^ portion, animals were subjected first to the hotplate test, immediately followed by the tail flick test. Disposable insulin syringes of 1 ml capacity with 29 gauge needles were used to deliver the 0.2 ml dose drop wise on to the scalp of the animals.

### Evaluation of antinociceptive effects:

Thirty six male rats were randomly divided into six groups. The rats in group 1 were treated orally with 1 ml of distilled water, group 2 with oral methadone hydrochloride aqueous solution 316.5 μg/ml, group 3 with 0.2 ml of sesame oil as the transcranial blank vehicle and groups 4, 5 and 6 with three different test dose levels of transcranial methadone oil solutions of 41.6 μg/0.2 ml as the low dose, 104 μg/0.2 ml as the mid dose and 208 μg/0.2 ml as the high dose, respectively.

The reaction time taken to experience the pain in seconds by the six rats in each group was determined using the hot plate and tail flick techniques[6]. The animals were screened one hour before treatment to select responsive animals having average normal base level reaction times of 7 - 8.5 s for the hot plate and 1.0-1.3 s for the tail flick methods at standard instrument settings. These normal base level reaction times were considered as the zero hour reaction times for the experiments. The animals were subjected to hot plate and tail flick tests at time intervals of 1, 2, 3, 4, 5 and 6 h after treatment. Time periods for groups 1 and 2 commenced from the time of feeding the dose and for groups 3, 4, 5 and 6 from the time of applying the first 1/3^rd^ portion of the transcranial formulation. In the hot plate technique, the rat was placed on an enclosed hot plate (model MK 35A, Muromachi Kikai Co. Ltd., Tokyo, Japan) at 50° and the reaction time taken to lick the hind paw was noted. In the tail flick technique, the tail of the rat was immersed 5 cm from the tip in a water bath maintained at 55° and the reaction time taken to flick the tail was noted. A cut-off time for the hot plate reaction time was set as 20 s while for the tail flick it was set at 10 s[7] to avoid injury to the animals.

### Statistical analysis:

The results were expressed as means±SEM. The statistical analyses were made using Mann-Whitney *U*-test. *P* values ≤ 0.05 were considered as significant.

## RESULTS AND DISCUSSION

**Fig. 1:**
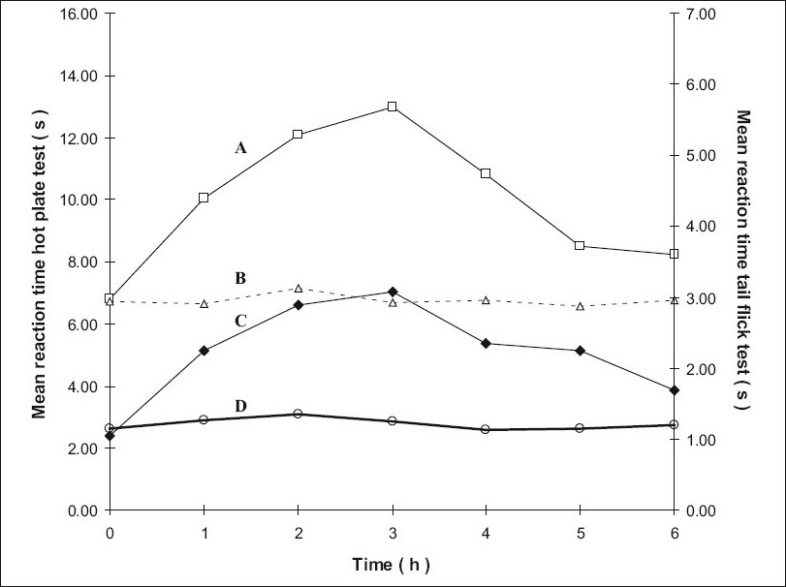
Antinociceptive effect with time in the hotplate and tail flick tests for groups 1 and 2. Left y-axis is for the curves A and B and right y-axis for the curves C and D. Curve A represents hot plate test results of group 2 treated with oral methadone (–□–) and Curve B group 1 control treated with distilled water (…Δ…), Curve C represents tail flick test results of group 2 treated with oral methadone (–◆–) and Curve D control of group 1 treated with distilled water (–○–).

**Fig. 2:**
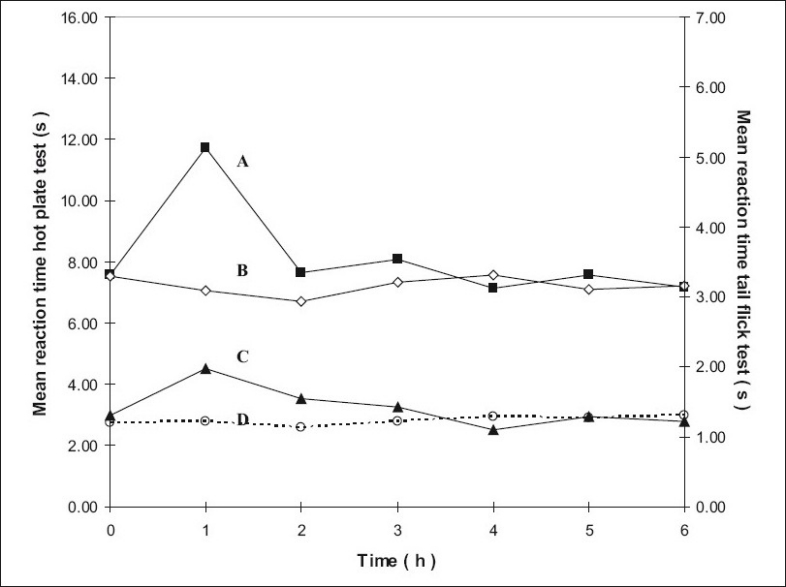
Antinociceptive effects with time using the hotplate and tail flick tests for groups 3 and 4. Left y-axis is for the curves A and B and right y-axis for curves C and D. Curve A represents hot plate test results of group 4 treated with transcranial methadone (–■–) and Curve B group 3 control treated with transcranial blank oil (–◊–), Curve C represents tail flick test results of group 4 treated with transcranial methadone (–▲–) and Curve D control of group 3 treated with transcranial blank oil (…○…).

**Fig. 3:**
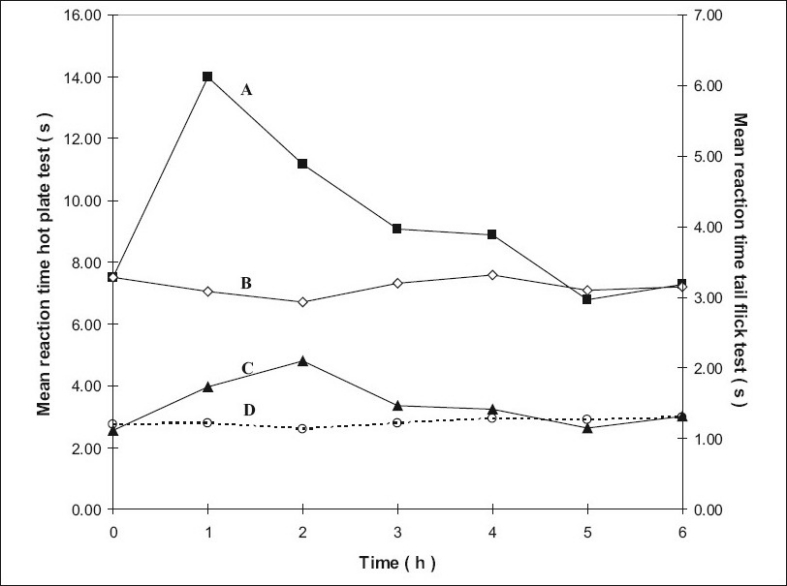
Antinociceptive effects with time using the hotplate and tail flick tests for groups 3 and 5. Left y-axis is for the curves A and B and right y-axis for curves C and D. Curve A represents hot plate test result of group 5 treated with transcranial methadone mid test dose (–■–) and Curve B represents group 3 control treated with transcranial blank oil (–◊–), Curve C represents tail flick test results of group 5 treated with transcranial methadone mid test dose (–▲–) and Curve D control of group 3 treated with transcranial blank oil (…○…).

**Fig. 4:**
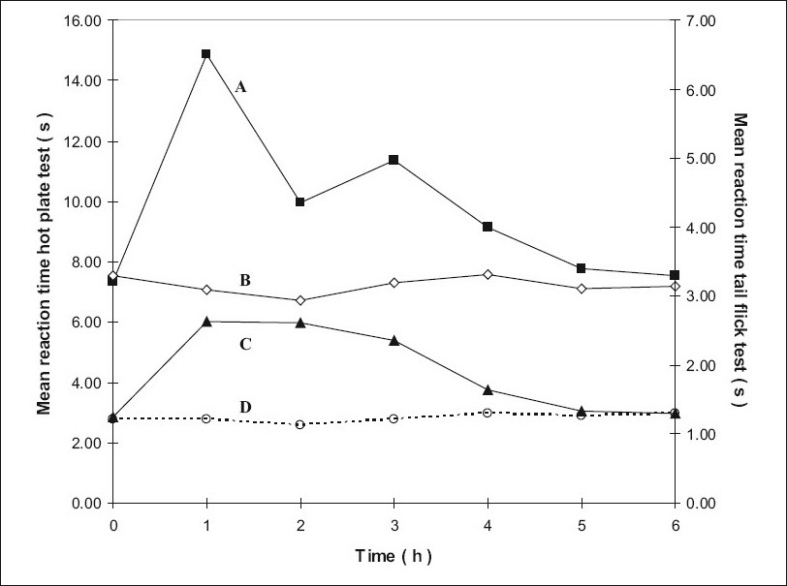
Antinociceptive effects with time of the hotplate and tail flick tests for groups 3 and 6. Left y-axis is for the curves A and B and right y-axis for curves C and D. Curve A represents hot plate test result of group 6 treated with transcranial methadone high test dose (–■–) and Curve B represents group 3 control treated with transcranial blank oil (–◊–), Curve C represents tail flick test results of group 6 treated with transcranial methadone high test dose (–▲–) and Curve D control of group 3 treated with transcranial blank oil (…○…).

The mean reaction times ± SEM of rats in the hotplate tests at different time intervals are summarized in Table 1 and the mean reaction times ± SEM in the tail flick tests at different time intervals are summarized in Table 2. The mean values were subjected to Mann-Whitney *U*-test and the differences were considered significant when P values were less than 0.05. The results are shown in figs. 1–4, as response vs time curves for the hot plate test on the left hand side y axis and the tail flick test on the right hand side y axis.

**TABLE 1:** MEAN REACTION TIME OF THE HOT PLATE TEST IN THE SIX GROUPS OF RATS WITH DIFFERENT TREATMENTS

Group	Mean reaction time ( s ) ± SEM						
	
	0 h	1 h	2 h	3 h	4 h	5 h	6 h
Group 1	6.72±0.22	6.63±0.36	7.17±0.26	6.70±0.27	6.75±0.31	6.58±0.32	6.75±0.22
Group 2	6.82±0.26	10.07±0.12	12.1±0.45	12.97±0.24	10.82±0.45	8.50±0.65	8.23±0.49
Group 3	7.52±0.20	7.05±0.32	6.72±0.51	7.32±0.24	7.58±0.32	7.10±0.36	7.20±0.24
Group 4	7.58+0.47	11.73±0.52	7.65±0.61	8.07±0.47	7.13±0.27	7.55±0.49	7.17±0.43
Group 5	7.50±0.49	14.00±1.35	11.15±1.76	9.05±1.01	8.87±0.65	6.78±0.42	7.28±0.76
Group 6	7.35±0.21	14.85±1.58	9.93±0.61	11.37±1.12	9.13±1.06	7.75±0.30	7.53±0.40

Group 1: treated with 1 ml distilled water (control), group 2: treated with oral methadone 316.5 μg/ml, group 3: treated with transcranial blank oil 0.2 ml (control), group 4: treated with transcranial methadone low test dose 41.6 μg/0.2 ml, group 5: treated with transcranial methadone mid test dose 104 μg/0.2 ml, group 6: treated with transcranial methadone high test dose 208 μg/0.2 ml. Each value is mean reaction time in seconds±SEM

**TABLE 2:** MEAN REACTION TIME OF THE TAIL FLICK TEST IN THE SIX GROUPS OF RATS WITH DIFFERENT TREATMENTS

Group	Mean reaction time ( s ) ± SEM						
	
	0 h	1 h	2 h	3 h	4 h	5 h	6 h
Group 1	1.15±0.05	1.27±0.05	1.35±0.14	1.25±0.09	1.13±0.08	1.16±0.07	1.20±0.05
Group 2	1.05±0.04	2.26±0.08	2.89±0.16	3.10±0.16	2.35±0.29	2.24±0.17	1.69±0.09
Group 3	1.21±0.07	1.21±0.09	1.13±0.04	1.22±0.08	1.29±0.07	1.27±0.06	1.30±0.11
Group 4	1.31±0.14	1.98±0.14	1.54±0.13	1.42±0.09	1.09±0.03	1.30±0.12	1.28±0.06
Group 5	1.12+0.12	1.73±0.24	2.10±0.28	1.46±0.23	1.42±0.09	1.15±0.08	1.31±0.13
Group 6	1.25+0.06	2.63±0.09	2.62±0.30	2.36±0.22	1.65±0.21	1.33±0.08	1.30±0.09

Group 1: treated with 1 ml distilled water (control), group 2: treated with oral methadone 316.5 μg/ml, group 3: treated with transcranial blank oil 0.2 ml (control), group 4: treated with transcranial methadone low test dose 41.6 μg/0.2 ml, group 5: treated with transcranial methadone mid test dose 104 μg/0.2 ml, group 6: treated with transcranial methadone high test dose 208 μg/0.2 ml. Each value is mean reaction time in seconds±SEM

Group 2 which was treated with oral administration of methadone hydrochloride 316.5 μg/ml shows a significant prolongation of the reaction time in the hot plate and tail flick tests from the first hour up to the sixth hour compared to the oral control group 1 treated with distilled water (fig. 1). When the group 4 animals were treated with 41.6 μg/0.2 ml low test dose of transcranial methadone solution, a significant analgesic effect was seen only in the first hour after treatment in the hot plate test, while the effect was significant in both the first and second hours in the tail flick test compared to control group 3 treated with 0.2 ml of transcranial blank oil (fig. 2). When compared to the blank oil control group 3, the 104 μg/0.2 ml mid test dose in group 5 exerted significant antinociceptive effects in the first and second hours after the transcranial treatment in the hot plate test as well as in the tail flick test (fig. 3). A similar significant antinociceptive effect was observed in group 6 following the 208 μg/0.2 ml high test dose transcranial methadone application in the first 3 h after treatment in the hot plate test and first 4 h after treatment in the tail flick test compared with the transcranial blank oil control group 3 (fig. 4). There was no significant difference between the oral negative control group 1 and the transcranial blank oil control group 3 in both hot plate and tail flick tests.

The results indicate a significant analgesic effect compared to transcranial blank oil control group 3 when methadone base was applied transcranially after dissolving in sesame oil. The highest test dose 208 μg/0.2 ml of the transcranial formulation markedly increased the reaction times in both the hot plate and tail flick tests. Groups treated with the transcranial formulation showed a rapid onset of activity in general. The analgesic activity of the highest dose and the mid dose of methadone was comparable to that of the oral positive control group 2 but the time of the peak activity was different between the two routes of administration. When the oral and the transcranial routes were compared it was observed that the duration of action by the oral route is longer than that of the transcranial route. An important observation in five out of the six curves in groups 4, 5 and 6 that were subjected to transcranial treatment is that the response vs time curves resemble those resulting after i.v. administration if the 00.00 h is not considered.

The results indicate that it is possible to deliver central nervous system drugs through the brain targeted transcranial route when applied on the scalp as oil solubilized dosage form. Systemic side effects of many common drugs could be overcome by the administration through the transcranial route since the drug targets the brain. In addition, the CNS side effects of drugs administered by other routes could also be over come by administering the specific antagonists through the new route. Further studies involving human volunteers may confirm the effectiveness of this new route of drug administration.
